# Targeting R-loops: diverse RNA helicases in R-loop resolution and their potential as targets for cancer therapy

**DOI:** 10.3389/fcell.2026.1822277

**Published:** 2026-04-21

**Authors:** Katherine Herrera, Kaoru Takasaki, Takahiko Murayama

**Affiliations:** 1 Department of Cell Biology, SUNY Downstate Health Sciences University, Brooklyn, NY, United States; 2 Division of Hematology, Department of Pediatrics, Children’s Hospital of Philadelphia, Philadelphia, PA, United States; 3 Perelman School of Medicine, University of Pennsylvania, Philadelphia, PA, United States

**Keywords:** cancer therapy, DEAD/DExH-box, replication stress, R-loop, RNA helicase

## Abstract

RNA helicases are enzymes that remodel RNA secondary structures and RNA-protein complexes using ATP-driven motor forces. They are known to participate in many essential cellular processes, including transcription, splicing, translation, RNA decay, and ribosome assembly. However, the functional diversity of RNA helicases and the multitude of associated cofactors make it difficult to grasp the full picture of their roles in these processes. Here, we focus exclusively on the R-loop-unwinding activities of RNA helicases and the cofactors involved in this process. R-loops are three-stranded nucleic acid structures that are mainly formed during transcription between newly synthesized mRNA and its template DNA. Timely resolution of R-loops by RNA helicases is required to prevent the DNA damage and replication stress that can result from collisions between transcription and DNA replication machinery acting aberrantly on the same DNA strand. Although R-loop resolution is critical for genome stability and cell proliferation, our understanding of the responsible helicases and their mechanisms remains incomplete. In this review, we summarize recent findings on R-loop-resolving helicases, discuss key questions and approaches for future investigation, and consider the potential of targeting these helicases for cancer therapy.

## Introduction

RNA helicases are ubiquitous enzymes that resolve RNA secondary structures (double-stranded (ds)RNAs, DNA/RNA hybrids, and more complex structures) and reconfigure RNA-protein complexes, which are involved in nearly all aspects of RNA metabolism including transcription, splicing, translation, RNA export and degradation, and ribosome biogenesis (Sloan and Bohnsack, 2018; Lang et al., 2024; Linder and Jankowsky, 2011; Jarmoskaite and Russell, 2014; Leppek et al., 2018). Helicases are classified into six superfamilies (SFs; SF1 - SF6) based on sequence, structure, and function. Eukaryotic RNA helicases belong to SF1 and SF2, which contain DEAD-box and DExH-box, the largest RNA helicase families in humans. (Jankowsky, 2011). These proteins share a conserved helicase core, consisting of RecA1 and RecA2 domains, but display a wide variety of accessory domains, substrate preferences, and mechanistic behavior (Lang et al., 2024). Human cells express roughly 70 RNA helicases, most of which are members of the SF2 superfamily (Sloan and Bohnsack, 2018); intriguingly, growing evidence indicates that these proteins are largely non-redundant. Recently, RNA helicases have attracted attention as guardians of genome stability by mitigating transcription-replication conflicts (TRC) and DNA damage by preventing harmful accumulation of R-loops (DNA/RNA hybrids) (Boleslavska et al., 2022; de Amorim et al., 2024; Sridhara et al., 2017; Dutrieux et al., 2025; Cargill M. et al., 2021). Thus, understanding how specific RNA helicases detect and unwind R-loops, and how different helicases contribute to R-loop resolution, is crucial for linking RNA metabolism to genome maintenance.

R-loops are three-stranded nucleic acid structures formed when a newly synthesized RNA strand hybridizes with its template DNA, displacing the non-template DNA strand (Niehrs and Luke, 2020). They arise naturally during transcription, DNA replication, and DNA repair (Cuartas and Gangwani, 2022). While R-loops can play regulatory roles, they can also be a source of genome instability when they accumulate at abnormally high levels (Niehrs and Luke, 2020; Petermann et al., 2022; Brickner et al., 2022; Garcia-Muse and Aguilera, 2019). Increasing evidence links unresolved R-loops to TRCs, DNA damage, and human disease, underscoring the need for dedicated resolution pathways (Garcia-Muse and Aguilera, 2019; Liu et al., 2025; Xu et al., 2024). RNA helicases have emerged as central factors in R-loop metabolism through ATP-dependent unwinding, since these enzymes mitigate R-loop accumulation across diverse organisms (Yang et al., 2023). This review focuses on RNA helicases implicated in R-loop unwinding, surveying biochemical activities, cellular phenotypes, and mechanistic models.

Furthermore, RNA helicases are frequently upregulated in many tumor types (Xie et al., 2022; Fuller-Pace, 2013; Robert and Pelletier, 2013), implying roles in tumor initiation, progression, and maintenance. If cancer cells depend on a specific helicase for survival, that helicase could be a promising therapeutic target. Indeed, several groups, including ours, have reported that genetic depletion of RNA helicases, including DDX5 (Ma et al., 2017; Du et al., 2017), DDX41 (Li et al., 2025; Hwang et al., 2024), and DHX9 (Cottrell et al., 2024; Cristini et al., 2018; Murayama et al., 2024), leads to cancer cell death and/or tumor growth suppression. Small-molecule inhibitors targeting these helicases have been developed (Ling et al., 2022; Li et al., 2023; Tentler et al., 2020; Yoneyama-Hirozane et al., 2017; Shinriki et al., 2022; Castro et al., 2024), and some of them are currently under clinical evaluation. Moreover, helicase inhibition can synergize with DNA damaging agents or replication stress inducers (Tao et al., 2026; Nicol et al., 2013; Chakraborty and Hiom, 2021; Liu M. Y. et al., 2024), selectively exploiting tumor-specific vulnerabilities in RNA processing and R-loop homeostasis, thus offering opportunities for combination therapies and biomarker-guided disease stratification. In this review, we first summarize the molecular mechanisms of R-loop resolution. Next, we introduce specific RNA helicases possessing R-loop unwinding ability, including both well-characterized factors and more recently implicated helicases. Lastly, we discuss available methodologies for detecting R-loops and helicase activity, highlight outstanding questions, and briefly explore their potential as therapeutic targets.

## Molecular mechanisms of R-loop unwinding

RNA helicases resolve R-loops mainly by harnessing ATP-driven unwinding to separate the RNA strand from the DNA/RNA hybrid. Members of the DEAD-box and DExH-box families commonly use either processive translocation or localized strand-separation to pull the RNA strand away from DNA, whereas Ski2-like and other helicases may engage extended substrates (Halbach et al., 2012; Johnson and Jackson, 2013). Recognition of a hybrid depends on structural cues such as the DNA-RNA junction, exposed single-stranded (ss)DNA, secondary structures in the RNA, and associated ribonucleoprotein complexes; these features may act as docking sites for helicases and their cofactors (Jankowsky, 2011; Cristini et al., 2018; Aguilera and Garcia-Muse, 2012). Helicases can also indirectly facilitate hybrid disruption by resolving or binding to G-quadruplexes (G4s) on the displaced strand, which stabilize R-loops (Santos-Pereira and Aguilera, 2015; Liu T. et al., 2024). Interactions with nascent RNA and transcription-elongation factors further target helicases to R-loops; for instance, Pérez-Calero and colleagues clearly showed that DDX39B colocalizes with nascent mRNA at promoters (Perez-Caler et al., 2020). In some cases (DDX1, DDX5, DDX18), the activity of poly(ADP-ribose) polymerase-1 (PARP1) contributes to helicase recruitment to R-loops. Lin et al. (2022) revealed that DHX9 is phosphorylated by serine/threonine-protein kinase ATR at S321, which is required for its association with R-loops under genotoxic stress (Liu M. Y. et al., 2024). These examples underscore the importance of protein-nucleic acid and protein-protein interactions in helicase recruitment.

Cofactors and post-translational modifications such as phosphorylation, ubiquitination, and methylation not only refine helicase recruitment but also activity at their targets, ensuring dynamic control of R-loop homeostasis (Jankowsky, 2011; Cristini et al., 2018; Liu M. Y. et al., 2024; Cargill M. J. et al., 2021; Mersaoui et al., 2019). The E3 ubiquitin ligase RNF39 regulates DDX3X stability via K48-linked ubiquitination (Wang et al., 2021). Protein arginine methyltransferase 5 (PRMT5) binds and methylates DDX5 at its RGG/RG motif, which is required for its interaction with the XRN2 exoribonuclease; although the methylation is not essential for DDX5 helicase enzymatic activity, PRMT5 deletion causes R-loop accumulation, indicating that cooperation between XRN2 and DDX5 is critical for effective hybrid resolution (Mersaoui et al., 2019). RNase H, which degrades the RNA strand, also often cooperates with helicases to prevent aberrant accumulation of R-loops; helicases can displace bound proteins or peel back RNA to expose the hybrid for RNase H-mediated cleavage (Aguilera and Garcia-Muse, 2012). Thus, the already-numerous helicases gain further functional diversity through distinct post-translational modifications and interactions with cofactors, and the specific functions of each helicase in R-loop resolution remain under active study.

**FIGURE 1:**
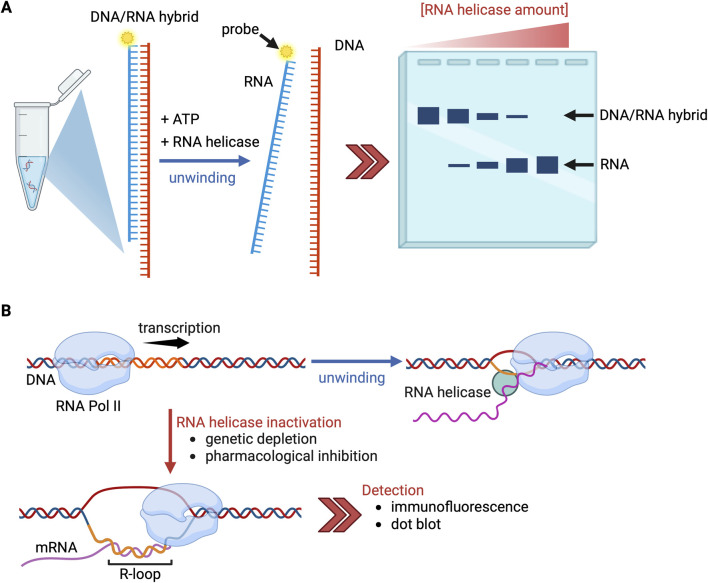
Experimental assays for R-loop resolution. **(A)** *In vitro* helicase assays; purified RNA helicases are incubated with DNA/RNA hybrid or R-loop model substrates in the presence of ATP. Unwinding is measured by changes in mobility of nucleic acids by gel electrophoresis. If RNA helicases contribute to R-loop resolution, the amount of DNA/RNA hybrid decreases as the amount of added RNA helicase increases. **(B)** Cellular assays; R-loop accumulation after depletion of helicase genes is detected using immunofluorescence and/or dot blot. Created with BioRender.com.

A standard approach to examine R-loop resolution is *in vitro* helicase assays, in which a DNA/RNA hybrid or R-loop substrate is incubated with ATP and recombinant helicase proteins (Figure 1A). Successful unwinding is detected as increased mobility of the probe RNA or DNA species on gel electrophoresis compared with the original hybrid. In cells, researchers commonly detect R-loops using the S9.6 antibody or inactive RNase H in immunofluorescence or dot blot (Skourti-Stathaki, 2022) (Figure 1B). These common approaches are powerful and convenient, but imperfect. One technical limitation is that the S9.6 antibody can cross-react with dsRNA (Hartono et al., 2018), so RNase H-treated controls are essential to confirm that the signal is derived from DNA/RNA hybrids. More importantly, RNA helicases are involved in multiple steps of RNA processing, all of which impact R-loop accumulation; S9.6 antibody cannot distinguish whether R-loop detection after helicase depletion reflects a direct loss of DNA/RNA hybrid-unwinding activity or a secondary consequence of impaired splicing or mRNA export. Nascent mRNA is detached from the template DNA after transcription, processed by the spliceosome, and then exported to the cytoplasm; because these steps are coordinated, defects in any of the factors involved in this pathway can dynamically induce R-loop accumulation. For example, Olazabal-Herrero and colleagues recently reported that defects in the splicing factor SRSF1, which interacts with the spliceosomal helicase DDX23 (Segovia et al., 2024), causes R-loop accumulation through impaired mRNA export (Olazabal-Herrero et al., 2024). It is thus crucial to dissect how R-loop accumulation is caused in helicase-depleted cells. One way to separate direct from indirect effects is to include knockdown controls of spliceosomal or mRNA export components. Sridhara et al. showed that depletion of DDX23, but not of its interacting splicing factors PRP8 and PRP6, increases R-loops, thus demonstrating a direct effect (Sridhara et al., 2017). Yet even with such controls, with current methods it remains difficult to determine definitively whether a helicase directly impacts R-loop accumulation.

In summary, helicases interact with cofactors to undergo distinct post-translational modifications and R-loops. Notably, although many helicases can unwind DNA/RNA hybrids *in vitro*, R-loop accumulation caused by knockdown of one helicase is usually not rescued by overexpression of another. This functional diversity of individual helicases likely arises from differences in cofactor-binding domains that confer context-dependent enzymatic activity. Despite the significant advances made in the field with technologies such as S9.6 antibody, open questions remain as to why cells need a diverse set of apparently nonredundant helicases, with specific topics such as the molecular determinants of substrate specificity and how helicases are prioritized during transcription-replication conflicts. Integrating proteomics with locus-resolved interaction mapping will be useful to define the networks that determine how individual helicases resolve R-loops. In the next section, we summarize RNA helicases that have been shown to resolve R-loops through their helicase activity (Table 1).

**TABLE 1:** Summary of R-loop resolution assay results and available inhibitors.

Gene	Cellular assays	*In vitro* assays	Inhibitors
SETX	+ (ref (Sberna et al., 2025; Crossley et al., 2023))	+ (ref (Hasanova et al., 2023))	-
AQR	+ (ref (Sollier et al., 2014; Sakasai et al., 2017))	+ (ref (Penzo et al., 2025))	-
DDX1	+ (ref (Ferri et al., 2025))	+ (ref (Moore et al., 2025))	-
DDX3X	+ (ref (Secchi et al., 2024))	+ (ref (Secchi et al., 2024; He et al., 2024))	+ (ref (Winnard et al., 2024))
DDX5	+ (ref (Mersaoui et al., 2019; Kang et al., 2021; Saha et al., 2022; Polenkowski et al., 2023))	+ (ref (Mersaoui et al., 2019))	+ (ref (Capasso et al., 2019; Diamond et al., 2017))
DDX17	+ (ref (Polenkowski et al., 2023))	+ (ref (Boleslavska et al., 2022))	-
DDX19	+ (ref (Hodroj et al., 2017a))	+ (ref (Hodroj et al., 2017a))	-
DDX21	+ (ref (Song et al., 2017; Hao et al., 2024))	+ (ref (Song et al., 2017))	-
DDX23	+ (ref (Sridhara et al., 2017))	- (ref (Segovia et al., 2024))	-
DDX39B	+ (ref (Perez-Caler et al., 2020))	+ (ref (Perez-Caler et al., 2020))	-
DDX41	+ (ref (Mosler et al., 2021; Weinreb et al., 2021))	+ (ref (Mosler et al., 2021))	+ (ref (Yoneyama-Hirozane et al., 2017))
DHX9	+ (ref (Cristini et al., 2018; Murayama et al., 2024))	+ (ref (Chakraborty and Grosse, 2011))	+ (ref (Castro et al., 2024))
SNRNP200	+ (ref (Chen et al., 2025))	+ (ref (Chen et al., 2025))	-
WRN	+ (ref (Marabitti et al., 2019))	+ (ref (Chakraborty and Grosse, 2010))	+ (ref (Picco et al., 2024; Baltgalvis et al., 2024; Ferretti et al., 2024))

## Specific RNA helicases with R-loop unwinding ability

### SF1 superfamily

#### SETX (senataxin)

Sen1, the yeast homolog of SETX, was the first reported R-loop helicase that unwinds the DNA/RNA hybrid structure (Mischo et al., 2011). In 2011, Mischo and colleagues found that loss of Sen1 in *S. cerevisiae* results in transient accumulation of R-loops, leading to activation of the DNA damage repair pathway (Mischo et al., 2011). In the same year, a very similar phenotype was observed in SETX-depleted human cells by Skourti-Stathaki *et al*; they reported that nascent transcripts form DNA/RNA hybrids behind elongating Pol II and that SETX is needed to resolve these structures (Skourti-Stathaki et al., 2011). *In vitro* analysis directly shows that SETX unwinds the DNA/RNA hybrid with a 5′-to-3′ polarity, using ATP as the energy source (Hasanova et al., 2023). Recently, it has been revealed that in cells with a high replication stress level, caused by oncogene *Myc* activation, *SETX* silencing leads to the accumulation of R-loops and TRC-derived DNA damage (Sberna et al., 2025), further supporting the idea that R-loop unwinding by this protein is important to prevent aberrant R-loop accumulation. R-loop accumulation caused by SETX depletion leads to cytoplasmic accumulation of DNA/RNA hybrids, which in turn can activate the innate immune system (Crossley et al., 2023).

#### AQR (aquarius)

AQR is a multifunctional putative RNA helicase (Beusch and Madhani, 2024). In 2014, Sollier et al. reported that knockdown of *AQR* in HeLa cells leads to the accumulation of R-loops and DNA damage, and that this phenotype was rescued by RNase H1 overexpression (Sollier et al., 2014). A similar phenotype was reported by another group showing that AQR knockdown impaired the DNA damage repair pathway, mediated by a double-strand break (DSB) processing factor CtIP (Sakasai et al., 2017). De and colleagues reported that recombinant AQR hydrolyzes ATP when it is stimulated by RNA and that it exhibits 3′-to-5′ dsRNA-unwinding activity (De et al., 2015). More recently, AQR helicase activity against DNA/RNA hybrids was confirmed by *in vitro* analysis using purified AQR protein (Penzo et al., 2025).

### SF2 superfamily

#### DDX1

DDX1 is a DEAD-box helicase involved in various cellular processes. Li and colleagues reported that DDX1 is required to remove RNA strands from the R-loops formed at sites of DSBs during the repair process (Li et al., 2016). Ferri et al. showed that siRNA knockdown of DDX1 leads to increased R-loop quantity and impaired Pol II-mediated transcription (Ferri et al., 2025), further supporting the idea that DDX1 is one of the critical regulators of R-loop resolution. Importantly, another group found that DDX1 binds to G4 structures and unwinds them into R-loops (Ribeiro de Almeida et al., 2018). *In vitro*, the ATP hydrolysis activity of DDX1 was confirmed by Moore and colleagues, who demonstrated that ssRNA molecules (as short as ten nucleotides), dsRNA, and DNA/RNA hybrids stimulated DDX1’s ATPase activity more strongly than ssDNA (Moore et al., 2025).

#### DDX3X

DDX3X is a gene on the X chromosome involved in many aspects of RNA metabolism (Mo et al., 2021). In human cells, the accumulation of R-loops was observed upon siRNA-mediated depletion of DDX3X (Secchi et al., 2024). The authors also confirmed the R-loop unwinding activity of DDX3X *in vitro* and suggested a role for DDX3X as an auxiliary factor for RNase H2 (Secchi et al., 2024). He et al. also reported that *in vitro*, DDX3X is a bi-directional RNA helicase that can directly unwind dsRNA and DNA/RNA hybrids in an ATP-dependent manner (He et al., 2024). Intriguingly, some inhibitors have been developed against DDX3X, since its high expression level correlates with poor overall survival in various cancers (Mo et al., 2021). RK-33, a small molecule inhibitor of DDX3, suppressed breast cancer bone metastases in a mouse model, suggesting that this molecule could be a promising target (Winnard et al., 2024).

#### DDX5

DDX5 is one of the most well-studied RNA helicases. In *in vitro* assays, DDX5 unwinds DNA/RNA hybrids, and conversely siDDX5 treatment leads to an accumulation of R-loops in cells (Mersaoui et al., 2019). Other groups have similarly reported that DDX5 is involved in the resolution of R-loops in other cell lines (Kang et al., 2021; Saha et al., 2022; Polenkowski et al., 2023).

DDX5 activity is known to be regulated by arginine methylation by PRMT5 (Mersaoui et al., 2019); however, Villarreal and colleagues reported that DDX5-depleted cells have unique R-loop gain peaks near transcription start sites that did not overlap with those of PRMT5-depleted cells, suggesting that DDX5 regulation is more complex (Villarreal et al., 2020). Recently, inhibitors targeting the DDX5 protein have been developed. For example, RX-5902, the first-in-class small molecule inhibitor of phosphorylated DDX5, efficiently induces apoptosis and G2/M cell-cycle arrest, thus inhibiting cellular proliferation *in vitro* (Capasso et al., 2019). The activity of the inhibitor was confirmed using *in vivo* models of triple-negative breast cancer, followed by a first-in-human phase I dose escalation study (NCT02003092); encouragingly, RX-5902 had a favorable side-effect profile in the subjects (Diamond et al., 2017).

#### DDX17

*In vitro* analyses directly show that DDX17 possesses helicase enzymatic ability against dsRNAs (Lee, 2002) and DNA/RNA hybrids (Boleslavska et al., 2022). Polenkowski et al. confirmed the DNA/RNA hybrid unwinding roles of both DDX17 and DDX5; they observed R-loop accumulation in DDX17-depleted and DDX5-depleted cells (Polenkowski et al., 2023). Furthermore, they found that overexpression of DDX17 or DDX5 suppresses R-loop accumulation caused by depletion of THO complex subunit 5 (THOC5), a member of the mRNA export complex, in HEK293 cells (Polenkowski et al., 2023), indicating that THOC5 itself is not involved in R-loop unwinding but is important for recruiting these helicases to the transcription machinery. On the other hand, Bader and colleagues reported that DDX17 promotes the formation of DNA/RNA hybrids at DSB sites (Bader et al., 2022), which suggests that DDX17’s function may differ under specific conditions, such as during DNA repair.

#### DDX19

DDX19, one of the DDX helicase family members, has been known to contribute to mRNA export out of the nucleus, which requires helicase enzymatic activity against RNA duplex structures (Schmitt et al., 1999). More recently, *in vitro* analysis by Hodroj et al. directly showed that recombinant wild-type DDX19, but not E243Q helicase-dead mutant, unwinds DNA/RNA hybrids (Hodroj et al., 2017a; Hodroj et al., 2017b). In addition, the authors showed that DDX19 knockdown induces R-loop accumulation and global DNA damage in HeLa cells (Hodroj et al., 2017a). In 2021, another group identified DDX19 as a coregulator of lysine-specific histone demethylase 1 (LSD1) activity and demonstrated that DDX19’s R-loop unwinding is necessary for the expression of actively transcribed genes (Pinter et al., 2021).

#### DDX21

Song and colleagues reported that recombinant DDX21 unwinds both dsRNA and DNA/RNA hybrid structures in an ATP-dependent manner (Song et al., 2017). In the same paper, the authors showed that knockdown of DDX21 leads to R-loop accumulation and DNA damage in MCF7 cells and that DDX21’s helicase activity is regulated by acetylation (Song et al., 2017). Additionally, Hao et al. identified DDX21 as a factor associated with m6A modification through a co-immunoprecipitation assay followed by mass spectrometry. They reported that DDX21’s R-loop helicase activity is necessary for methyltransferase-like 3 (METTL3)-mediated m6A deposition onto nascent RNA, which regulates transcription termination (Hao et al., 2024), further supporting the idea that DDX21 is one of the crucial factors for R-loop resolution during transcription.

#### DDX23

DDX23 is a DDX helicase that has been identified as part of the spliceosomal U5 small nuclear ribonucleoprotein (U5 snRNP) (Teigelkamp et al., 1997; Wood et al., 2021). Sridhara et al. reported that DDX23 knockdown induces the accumulation of R-loops and DNA damage in U-2OS cells (Sridhara et al., 2017). The authors also showed that spliceosome activity is not necessary for the suppression of DNA damage by DDX23, since depletion of PRP8 or PRP6, which are core components of U5 snRNP, does not induce DNA damage in the cells. In addition, Ruan and colleagues reported that DDX23 directly binds to synthetic dsRNA (Ruan et al., 2019). However, since both human DDX23 and yeast Prp28 have very low RNA-dependent ATPase activity and their presumed RNA helicase activity has never been detected in *in vitro* helicase assays (Segovia et al., 2024), it is still unclear whether DDX23 directly unwinds DNA/RNA hybrids or indirectly contributes to R-loop resolution.

#### DDX39B

The DEAD-box helicase DDX39B (or UAP56) is a known component of the conserved TRanscription-EXport (TREX) complex, which is involved in multiple steps of gene expression (Bhattacharjee et al., 2025). Pérez-Calero et al. found that purified wild-type DDX39B protein, but not K95A or E197A mutants, unwinds DNA/RNA hybrids *in vitro* (Perez-Caler et al., 2020). The authors also reported that siRNA-mediated depletion of DDX39B causes R-loop accumulation and DNA damage in HeLa cells (Perez-Caler et al., 2020). Importantly, overexpression of DDX39B reduced R-loop levels and DNA damage caused by depletion of other helicases, including DDX23, SETX, and AQR (Perez-Caler et al., 2020), suggesting that there is, at least partially, functional redundancy among certain RNA helicases.

#### DDX41

*In vitro* experiments by Mosler et al. demonstrated that recombinant DDX41 can unwind DNA/RNA hybrids, but the helicase domain-lacking mutant cannot (Mosler et al., 2021). The authors also showed that DDX41 depletion leads to the accumulation of R-loops and DNA damage in U-2OS cells (Mosler et al., 2021). Additionally, a recent *in vitro* study by Bi and colleagues showed that DDX41 directly binds to and resolves G4 structures (Bi et al., 2025), which also contributes to the maintenance of genome stability. Interestingly, DDX41 mutations are the most common germline predisposition to myelodysplastic syndrome (MDS) and acute myeloid leukemia (AML) (Sebert et al., 2019; Li et al., 2022; Makishima et al., 2023). Although cases of MDS with germline truncating mutations in DDX41 rapidly progress to AML (Makishima et al., 2023), they appear to have a better overall survival compared to wild-type DDX41 cases, suggesting a distinct mechanism of leukemic transformation caused by DDX41 deficiency (Li et al., 2022; Makishima et al., 2023). Although it is still unclear whether the accumulated R-loops are a major driver of AML, Weinreb et al. reported that elevated R-loop accumulation in DDX41-mutated human hematopoietic stem and progenitor cells (HSPCs) triggers inflammatory pathway activation and HSPC overproduction (Weinreb et al., 2021), further supporting the idea that DDX41 is critical for hematopoietic homeostasis. Conversely, DDX41-selective inhibitors identified by Yoneyama-Hirozane and colleagues (Yoneyama-Hirozane et al., 2017) have been reported to efficiently induce R-loop accumulation and DNA damage in HeLa and K562 cells (Shinriki et al., 2022).

#### DHX9

DHX9 can unwind DNA/RNA hybrids, dsRNA, and dsDNA structures (more efficiently on RNA-containing duplexes) with a 3′–5′ polarity (Chakraborty and Grosse, 2011). Cristini and colleagues reported that concurrent camptothecin treatment and DHX9 depletion trigger R-loop accumulation and DNA damage in HeLa cells (Cristini et al., 2018). Several groups, including ours, reported that DHX9 depletion can induce immune responses through accumulation of cytoplasmic dsRNA and/or dsDNA (Cottrell et al., 2024; Murayama et al., 2024; Ren et al., 2024). Importantly, a first-in-class inhibitor was developed by Accent Therapeutics, and they have found that the pharmaceutical inhibition of DHX9 can efficiently kill genome-unstable cancer cells (Castro et al., 2024).

#### SNRNP200

SNRNP200, a DExH-box family helicase, unwinds U4/U6 small nuclear (sn)RNAs, which are required for catalytic activation of the spliceosome (Zhao et al., 2009). Chen and colleagues showed that recombinant Brr2 (the yeast homolog of SNRNP200) can unwind DNA/RNA hybrid structures *in vitro* (Chen et al., 2025). They also reported that knockdown of Brr2 causes R-loop accumulation in cells and that overexpression of Brr2, but not a helicase-dead mutant, rescues R-loop accumulation caused by depletion of Hel25E (the yeast homolog of DDX39B) (Chen et al., 2025), suggesting that these proteins have some functional redundancy.

#### WRN (Werner)

Werner’s syndrome (WS) is a monogenic disease caused by mutations in the *WRN* gene (Chakraborty and Grosse, 2010; Oshima et al., 2017). Chakraborty and colleagues’ *in vitro* model showed that recombinant WRN can unwind DNA/RNA hybrid structures (Chakraborty and Grosse, 2010). Marabitti et al. compared WS (i.e., WRN-mutated) and WRN-corrected WS (i.e., WRN wild-type) cells to demonstrate that loss of functional WRN results in R-loop accumulation and DNA damage, suggesting that WRN is one of the important regulators of R-loops (Marabitti et al., 2019). Inhibitors targeting WRN have been developed (Picco et al., 2024; Baltgalvis et al., 2024; Ferretti et al., 2024): HRO761, a selective and allosteric WRN inhibitor, causes DNA damage and inhibition of tumor growth in preclinical models and is now being tested in a clinical trial (NCT058387680) (Ferretti et al., 2024).

## Discussion

In this review, we summarize recent findings on RNA helicases, focusing on their R-loop unwinding activity. Accumulating evidence indicates that many RNA helicases can directly unwind R-loops and that their deregulation leads to replication stress, DNA damage, and transcriptional defects (Niehrs and Luke, 2020; Yang et al., 2023). However, it remains unclear why cells express so many apparently non-redundant helicases. Reports in which R-loops formed after depletion of one helicase and were resolved by overexpression of another are relatively uncommon (many rescue experiments instead use RNase H1 overexpression), although some studies have reported partial functional overlap (Perez-Caler et al., 2020; Chen et al., 2025). Helicase activity is unlikely to be target sequence-dependent; rather, individual helicases may act in specific contexts (such as DNA repair, transcription initiation, elongation, or termination) or prefer hybrids of a particular length or structure. The experiments performed so far are insufficient to resolve these possibilities, and further investigation is needed.

A major obstacle to understanding mechanisms is the limited and context-dependent detection of R-loops. S9.6-based immunofluorescence or dot-blot assays often detect R-loop increases after helicase depletion (Skourti-Stathaki, 2022; Ramirez et al., 2021), but these results do not distinguish direct defects in hybrid-unwinding activity from indirect effects such as impaired splicing or RNA export. *In vitro* unwinding assays can test direct activity but may fail to recapitulate relevant cofactors and chromatin context. Investigating differences in R-loop profiling by DRIP-seq (based on S9.6 or catalytically inactive RNase H) and utilizing emerging technologies (such as *in vivo* single-molecule imaging or improved locus-specific R-loop mapping) should help clarify helicase specificity and mode of action.

Small-molecule inhibitors of RNA helicases have been developed and show encouraging activity in *in vitro* and preclinical models. Helicases are frequently overexpressed in cancers compared with normal tissues (Xie et al., 2022; Fuller-Pace, 2013), which may provide a favorable therapeutic window; however, the clinical evidence remains limited. Inhibitors targeting DDX3X and DDX41 are still at the preclinical stage or moving toward clinical trials, while DDX5, DHX9, and WRN inhibitors have or are being tested in phase I or phase I/II trials (Diamond et al., 2017; Picco et al., 2024; Ferretti et al., 2024). The DDX5 inhibitor RX-5902 was well-tolerated in a phase I study focused on relapsed/refractory solid tumors, with adverse events limited to grade 1–2 anorexia, nausea, vomiting, diarrhea, and fatigue in some patients (Diamond et al., 2017). The phase I/Ib clinical trial (NCT05838768) of HRO761, a WRN inhibitor, met its accrual as of February 2026, while a separate phase I study (NCT06004245) of another WRN inhibitor, VVD-133214, remains open. Despite these encouraging developments, rigorous validation of target specificity is critical, since broad inhibition of RNA helicases could cause toxicity by disrupting RNA processing in normal cells. Indeed, phase II clinical trial (NCT02003092) of the DDX5 inhibitor RX-5902 for triple-negative breast cancer was terminated for unspecified reasons. Identification of predictive biomarkers, including specific R-loop signatures, DNA repair defects, and mutation profiles, could help determine which cancer types can be good targets and enable the efficient and safe use of these inhibitors in cancer therapy.

In summary, although our knowledge on why cells need such various RNA helicases for R-loop resolution is still limited, these enzymes represent promising therapeutic targets, particularly in genome-unstable cancers. A deeper mechanistic understanding of R-loop unwinding will inform basic transcriptional biology and guide rational combinations that exploit helicase inhibition to selectively induce replication stress in tumors.
